# The effect of working memory load on interference inhibition in table tennis athletes: the moderating role of motor expertise

**DOI:** 10.3389/fpsyg.2026.1812346

**Published:** 2026-04-28

**Authors:** Hongyu Chen

**Affiliations:** College of Physical Education and Health, East China Normal University, Shanghai, China

**Keywords:** interference effect, interference inhibition, motor expertise, table tennis athletes, working memory load

## Abstract

**Background:**

Although the effect of working memory load on interference inhibition has been widely investigated, how the effect manifests in expert athletes remains unclear.

**Methods:**

A dual-task paradigm combining an n-back task and a Spatial Stroop task was employed. 20 table tennis athletes and 19 non-athletes were recruited, and event-related potentials (ERPs) and event-related spectral perturbations (ERSPs) were analyzed to examine underlying neural activity patterns.

**Results:**

In the Spatial Stroop task, increased working memory load reduced the interference effect on RTs and midfrontal theta power, and was associated with decreased parietal alpha power. Regarding the P300, increased working memory load reduced the interference effect on P300 amplitude in non-athletes. In contrast, the athletes demonstrated a lower interference effect on P300 amplitude and showed no significant impact of working memory load.

**Conclusion:**

Increased working memory load reduced the impact of distractors, thereby leading to an overall reduction in the Stroop interference effect. Motor expertise was found to moderate the effect of working memory load on interference inhibition, specifically at the P300 amplitude level. Compared with non-athletes, table tennis athletes exhibited a more efficient and stable pattern of attentional resource allocation under working memory load, this pattern suggests that the moderating role of motor expertise is component-specific. Taken together, the present findings extend existing research on working memory and interference inhibition in athletes and deepen our understanding the relationship between long-term specialized training and brain function.

## 1 Introduction

Inhibitory control refers to the ability to regulate one's attention, behavior, thoughts, or emotions to resist internal and external interference and engage in more appropriate or goal-directed actions (Diamond, 2013). Interference inhibition represents the cognitive aspect of inhibitory control and refers to the ability to suppress task-irrelevant conflicting information or distracting stimuli (Diamond, 2013; Miyake et al., 2000). This ability is commonly assessed using interference effect in the Flanker task (Eriksen and Eriksen, 1974) and the Stroop task (Stroop, 1935). In general, larger interference effect indicates greater susceptibility to distractors and is associated with weaker inhibitory control.

Recent studies have demonstrated that working memory load significantly influences individuals' ability to inhibit distractors (Brockhoff et al., 2022; Lavie et al., 2004; Li et al., 2025; Luna et al., 2020; Kim et al., 2005; Park et al., 2007; Scharinger et al., 2015; Sörqvist and Rönnberg, 2014). For example, according to the load theory of selective attention, increased working memory load can impair top-down cognitive control processes, impair inhibitory control processes, and result in greater interference effect in inhibitory control tasks (Lavie et al., 2004; Lavie and De Fockert, 2005; Wei and Zhou, 2020). In contrast, the task-engagement/distraction trade-off (TEDTOFF) model posits that working memory load serves as a “shield” against distractors, increasing task demands and enhancing attentional engagement, thereby reducing their impact (Sörqvist and Rönnberg, 2014; Sörqvist and Marsh, 2015). Consequently, this process can reduce interference effect in inhibitory control tasks (Bayramova et al., 2021; Güldenpenning et al., 2020; Luo et al., 2025). Neuroimaging and electrophysiological studies have further demonstrated that the attentional enhancement mechanism proposed by the TEDTOFF model is associated with efficient activation of fronto-parietal attentional network induced by working memory load. For example, an fMRI study by (Sörqvist et al. 2016) demonstrated that, during an n-back task combined with auditory stimuli, the 3-back condition reduced activities in brain regions involved in processing auditory distractors, while simultaneously eliciting robust activation in prefrontal and parietal cortices. (Scharinger et al. 2015) further reported that, compared with the 0-back condition, the 2-back condition significantly reduced interference effect on parietal P300 amplitude and alpha power during a Flanker task, possibly due to enhanced engagement of attentional networks and a reduced impact of flanker distractors. In line with this finding, (Kim et al. 2017) employed independent component analysis, source localization, and Granger causality analysis to demonstrate that increased n-back task demands enhanced causal connectivity within the fronto-parietal attentional network during the Flanker task, thereby facilitating more efficient integration of neural information.

Taken together, existing evidences on the effect of working memory load on interference inhibition remain inconsistent. However, compared with load theory, the TEDTOFF model places greater emphasis on the role of individual cognitive differences in this process. For example, previous studies have shown that individuals with higher working memory capacity are generally less susceptible to distractors across varying levels of cognitive load, suggesting a moderating role for domain-general cognitive ability (Hu et al., 2019; Marsh et al., 2015; Sörqvist and Rönnberg, 2014). However, individual cognitive differences are not limited to innate traits or domain-general abilities; long-term, deliberate training, as a powerful external factor, can reshape brain structure and functional system, thereby giving rise to domain-specific cognitive differences (Ullén et al., 2016). Therefore, it is necessary to examine how the TEDTOFF model operates in populations that have undergone long-term training and exhibit enhanced cognitive performance, in order to both deepen our understanding of training-induced cognitive differences and evaluate the model's generalizability.

In recent years, the interaction between sports and cognition has been discussed as a central research topic. Long-term, regular specialized training has been shown to induce significant enhancements in athletes' brain function and cognitive performance, giving rise to a motor expertise-based advantage (Peng et al., 2025; Ren et al., 2025; Ullén et al., 2016). An increasing body of empirical evidence suggests that long-term specialized training is associated with enhanced inhibitory control in athletes (Chen et al., 2019; Simonet et al., 2022, 2023; Vestberg et al., 2012, 2017; Voss et al., 2010). Moreover, athletes often exhibit lower levels of neural activation or a more economical use of neural resources while achieving comparable behavioral performance, a phenomenon commonly referred to as neural efficiency (Li and Smith, 2021; Neubauer and Fink, 2009). Empirical evidence from studies on table tennis athletes further supports this perspective. Specifically, compared with non-athletes, table tennis athletes have demonstrated faster reaction times and more efficient inhibitory release during inhibitory control tasks (Zhu et al., 2022), along with lower levels of prefrontal activation and reduced ERP component amplitudes (Guo et al., 2017; Wei and Li, 2017; Xu et al., 2022). (Huang et al. 2024) further reported that table tennis athletes exhibited reduced lower Ninc amplitude and reduced interference effect on the parietal LSP component in a Color–Word Stroop task, as well as reduced interference effect on reaction times and the parietal LSP component in a Spatial Stroop task. These findings suggest that table tennis athletes exhibit superior interference inhibition during the processing of both semantic and Spatial information. In summary, the existing evidences indicate that long-term specialized training leads table tennis athletes to exhibit enhanced inhibitory control and characteristics of neural efficiency, reflecting a domain-specific cognitive difference derived from motor expertise. However, the cognitive processing of table tennis athletes within the TEDTOFF framework remains unclear. Specifically, although table tennis athletes exhibit superior inhibitory control compared with non-athletes, it remains unclear whether their inhibitory performance is less susceptible to working memory load. In other words, does motor expertise moderate the effect of working memory load on interference inhibition?

To address this question, the present study recruited table tennis athletes and non-athletes (normal university students without racket-sport experience) as participants, thereby identifying the unique effect of motor expertise. Second, a dual-task paradigm combining an n-back task and a Spatial Stroop task was employed to assess working memory load and interference inhibition for the following reasons: (1) The TEDTOFF model, together with the findings of (Scharinger et al. 2015), suggests that future research should examine whether the phenomenon whereby working memory load reduces distractors can be extended to cognitively comparable task combinations, such as the n-back and Stroop tasks; (2) The arrow stimuli used in the Spatial Stroop task and the digits used in the n-back task are independent in terms of stimulus properties, thereby avoiding potential confounds arising from stimulus overlap when examining the effect of working memory load on interference inhibition (Kim et al., 2005; Park et al., 2007). In this context, the present study used the interference effect in the Spatial Stroop task (i.e., the Stroop interference effect), defined as the difference between performance indices under incongruent and congruent conditions, to index the impact of distractors on individual performance. Finally, electroencephalography (EEG) was employed to investigate underlying neural activity characteristics, with event-related potentials (ERPs) techniques providing millisecond-level temporal resolution for recording brain responses to time-locked events. The P300 is an ERP component that typically peaks over parietal regions between 300 and 600 ms following stimulus onset. When individuals process conflicting information or distractors, longer P300 latency and larger P300 amplitude are typically elicited, reflecting slower cognitive processing and increased allocation of attentional resources (Magliero et al., 1984; Polich and Kok, 1995; Polich, 2007; Wang et al., 2014; Yu et al., 2018; Zhang et al., 2019). Additionally, Event-related spectral perturbations (ERSPs) techniques capture a broader range of non-phase-locked neural activities by examining frequency-specific power changes, thereby providing richer information about underlying cognitive processes. In tasks such as the Flanker and Stroop paradigms, incongruent conditions, relative to congruent conditions, reliably elicit increased midfrontal theta band power (4-7Hz), reflecting heightened demands for cognitive control during the conflict detection stage (Cavanagh and Frank, 2014; Haciahmet et al., 2023; Nigbur et al., 2012; Pastötter et al., 2013). Parietal alpha band is thought to reflect attentional gating mechanisms during information processing, such that reductions in alpha band power (8-13Hz) typically indicate inhibitory modulation of posterior brain regions that are not required for task (Klimesch et al., 2007; Klimesch, 2012).

In sum, we hypothesized that (1) in the non-athletes, increased working memory load would reduce the impact of distractors, as reflected by decreased Stroop interference effect at both behavioral and electrophysiological levels. (2) Whereas in the athletes, Stroop interference effect at both behavioral and electrophysiological levels were expected to be overall lower and less affected by working memory load, indicating a moderating role of motor expertise.

## 2 Materials and methods

### 2.1 Participants

This study recruited two groups of participants, athletes and non-athletes. Athletes were required to meet the following criteria: (1) being native Chinese speakers with normal or corrected-to-normal vision, right-handedness, and no history of neurological or psychological disorders; (2) holding a National Level 2 or higher table tennis athlete certification, having received intensive table tennis training within the past 3 months (with a training frequency of at least three sessions per week, each lasting 2–4 hours), and having participated in competitions up to the national level. non-athletes met the following criteria: (1) being native Chinese speakers with normal or corrected-to-normal vision, right-handedness, and no history of neurological or psychological disorders; and (2) being undergraduate students without prior training in table tennis or other racket sports and without any athlete-level certification. Sample size was determined using G^*^Power 3.1, assuming a correlation coefficient of 0.5 for repeated measures, an effect size of 0.25, a confidence level of 0.95, and a statistical power of 0.90, which indicated 30 participants. Finally, 39 participants were recruited, including 20 table tennis athletes (10 males and 10 females; mean age = 19.93 ± 0.83 years; mean training experience = 13.21 ± 1.21 years) and 19 non-athlete university students (9 males and 10 females; mean age = 20.42 ± 0.72 years). All participants signed informed consent forms after receiving a thorough explanation of the experimental procedures and received appropriate compensation upon completion of the experimental tasks. The experimental protocol for this study was approved by the Ethics Review Committee (Approval No.102772024RT094) and strictly adhered to the ethical standards outlined in the Declaration of Helsinki.

### 2.2 Materials

The experiments were designed and executed using E-Prime 3.0. The stimuli were displayed on a 30-inch Dell monitor with a resolution of 1980 × 1470 pixels and a refresh rate of 60 Hz. Participants were seated at an approximate viewing distance of 60 cm from the screen. Electroencephalographic (EEG) signals were recorded using a 64-channel Ag/AgCl electrode cap arranged according to the international 10–20 system, with a sampling rate of 1,000 Hz (Oostenveld and Praamstra, 2001). During data acquisition, the EEG signal was referenced online to the midpoint between Cz and CPz, with a common ground at the midpoint between FPz and Fz. EEG data were collected using the actiChamp system (Brain Products GmbH, Gilching, Germany), with electrode impedances maintained below 5 kΩ throughout the recording.

### 2.3 Procedures

The N-back task is frequently employed to assess working memory and its updating function (Niendam et al., 2012; Scharinger et al., 2017; Owen et al., 2005). This task requires participants to continuously update their memory content, responding only when the current stimulus matches the one presented n trials prior. The level of working memory load is manipulated by setting n. Following previous studies by (Scharinger et al. 2015) and (Kim et al. 2017), two load conditions were implemented: a low-load (0-back) and a high-load (2-back) condition. Task stimuli consisted of Arabic numerals (0–9) presented in Roman font. In the low-load (0-back) condition, participants responded to a predefined target digit, without any requirement to update information in memory. In the high-load (2-back) condition, participants continuously maintained the two most recent digits in memory and responded on each trial when the current digit matched the one presented two trials earlier.

The Spatial Stroop task was employed to assess interference inhibition. In this task, participants were required to judge the direction indicated by an arrow while ignoring interference arising from the arrow's spatial location (Tafuro et al., 2019; Zoccatelli et al., 2010). The stimuli consisted of horizontal black arrows (RGB: 0, 0, 0) with a visual angle of 1.91° × 2.87°, which were randomly presented 1.91° to the left or right of a central fixation point (0.95° × 0.95°), defined as the distance between the centers of the arrow and the fixation point. The task involves two conditions: a congruent condition, where the arrow direction aligns with its spatial position (e.g., a left-pointing arrow appears to the left of the fixation point); and an incongruent condition, where the arrow direction does not align with its spatial position (e.g., a right-pointing arrow appears to the left of the fixation point). Both conditions appear randomly in each trial with equal probability (50%).

**Figure 1:**
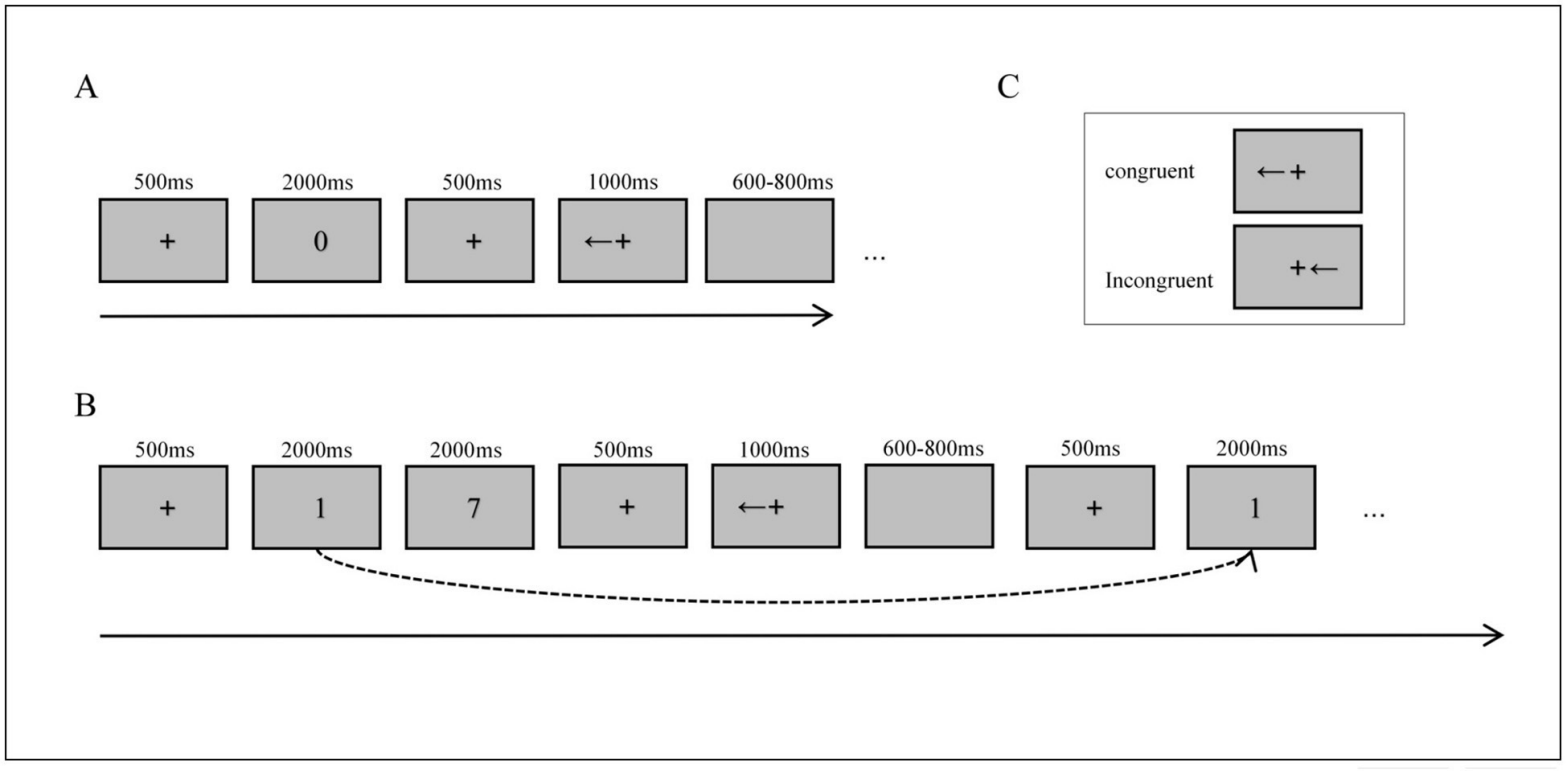
Schematic illustration of experimental trial structures and stimulus types. **(A)** Structure of the dual-task under low-load (0-back) condition. **(B)** Structure of the dual-task under high-load (2-back) condition. **(C)** Schematic of the Spatial Stroop task under congruent and incongruent conditions.

In the dual-task of this study, one trial comprised an n-back task and a Spatial Stroop task. For the low-load (0-back) condition, each trial comprised two phases. (1) Memory digit judgment phase: A black fixation point was presented for 500 ms, followed by the random presentation of a digit. Participants were instructed to press the “X” key with the left middle finger if the digit was 0, and the “C” key with the left index finger if the digit was not 0. (2) Interference inhibition task phase: Following a 500 ms fixation, a left- or right-pointing arrow appeared randomly to the left or right of the fixation point. Participants responded by pressing the “←” key with the right index finger for left-pointing arrows and the “ → ” key with the right middle finger for right-pointing arrows. The maximum response window was 1,000 ms; trials with no response were recorded as errors and were followed by a blank screen lasting 600–800 ms. For the high-load (2-back) condition, each trial consisted of three phases. (1) Memory encoding phase: After a 500 ms fixation, two digits were presented sequentially (2000 ms per digit; 4,000 ms total). During this phase, participants encoded the digits without making any response. (2) Interference inhibition task phase: Following a 500 ms fixation, a left- or right-pointing arrow appeared randomly to the left or right of the fixation point. Response mappings and timing constraints were identical to those in the 0-back condition, with a maximum response time of 1000 ms and non-responses recorded as error trials, followed by a 600–800 ms blank screen. (3) Memory digit detection and updating phase: After a 500 ms fixation, a probe digit was presented. Participants judged whether the probe digit matched the digit presented two positions earlier (i.e., the target stimulus for n = 2). If the digits matched, participants pressed the “X” key with the left middle finger; if they did not match, participants pressed the “C” key with the left index finger. Following the response, participants updated their working memory, as the probe digit served as the reference item for subsequent trials (see Figure 1).

Before the formal experiment, participants completed 20 practice trials under each working memory load condition to ensure that they fully understood the task procedures. In the formal experiment, all participants performed the dual-task paradigm under both load conditions. Each load condition consisted of 168 trials, which were divided into 7 blocks of 24 trials each. After completing each block, participants were presented with a rest screen and were instructed to resume the task by pressing the space bar when ready. The order of the load conditions was counterbalanced within participants: half completed the experiment in the low-load, then high-load order, whereas the remaining participants completed the conditions in the reverse order.

### 2.4 EEG recording and analysis

EEG data preprocessing was conducted using the EEGLAB toolbox (version 2020) implemented in MATLAB (R2020b). First, the EOG electrodes were removed, bad channels were interpolated from good channels, and the EEG data were re-referenced to the average of all scalp electrodes. Subsequently, the data were band-pass filtered with a high-pass cutoff at 0.1 Hz and a low-pass cutoff at 30 Hz; additionally, notch filters at 48 Hz and 52 Hz were applied to eliminate noise. As a next step, EEG epochs corresponding to the Spatial Stroop task were extracted from −200 ms to 1,000 ms relative to arrow stimulus onset, with the pre-stimulus interval (−200 to 0 ms) used for baseline correction in ERP analyses. Finally, independent component analysis (ICA) was applied to identify and remove components associated with eye blinks and movement-related artifacts. Finally, epochs with amplitudes exceeding ±100 μV were excluded, and trials with no response or incorrect responses were removed from further analyses.

### 2.5 Data analysis

The statistical analyses were performed using SPSS 27. The degrees of freedom were adjusted using the Greenhouse-Geisser correction, and *post hoc* tests were corrected for multiple comparisons as needed. Statistical significance was set at *p* < 0.05, and partial eta squared ($\eta_{p}^{2}$) was reported as the measure of effect size for the ANOVA. Finally, descriptive results are presented as the mean ± standard error (M ± SE).

#### 2.5.1 Behavioral analysis

Only trials that were correct in both the memory task and the Stroop task were included in all subsequent analyses. In addition, trials in which response times (RTs) deviated by more than three standard deviations from the condition mean were excluded from behavioral analyses.

#### 2.5.2 ERP analysis

Based on topographic map observations, the P300 component was analyzed within a 300–550 ms time window at parietal electrodes (P1, P2, and Pz), and the mean latencies and amplitudes were calculated across these electrodes.

#### 2.5.3 ERSP analysis

To avoid edge artifacts, EEG data were re-epoched from 500 ms to 1,000 ms relative to stimulus onset. The Continuous Morlet Wavelet Transform (Mouraux and Iannetti, 2008) was employed to perform time–frequency analysis of the EEG data. The center frequency parameter (ω) and smoothing parameter (σ) were set to 5 and 0.15, respectively. Time–frequency representations were computed over a frequency range of 1–30 Hz with a step size of 1 Hz. Single-trial time-frequency representations were obtained following wavelet transformation and were subsequently averaged across trials within each condition. Event-related spectral perturbations (ERSPs) were calculated at each time point and frequency to characterize sustained changes in spectral power following stimulus onset. ERSP values were computed using the formula ER_t, f_% = (A_t, f_ − R_*f*_)/R_*f*_, where A_t, f_ denotes signal energy at a given time point and frequency during task performance, and R_*f*_ represents the mean signal energy during the baseline interval (Pfurtscheller and Da Silva, 1999). The baseline period was defined as the interval from −400 to −200 ms preceding stimulus onset in the Spatial Stroop task.

The theta band was defined as 4–7 Hz, with a time window of 250–500 ms, and the mean power across three fronto-central electrodes (Fz, FCz, and Cz) was extracted. The alpha band was defined as 8–13 Hz, with a time window of 400–600 ms, and the mean power across three parietal electrodes (Pz, P3, and P4) was extracted.

## 3 Result

### 3.1 Behavioral results

#### 3.1.1 N-back task

A 2 (group) × 2 (working memory load) repeated-measures ANOVA was conducted on the RTs in the n-back task; the results revealed a significant main effect of working memory load, F_(1, 37)_ = 283.08, *p* < 0.001, η_*p*_^2^ = 0.87. RTs were significantly faster under the low-load condition (526.13 ± 4.78 ms) than under the high-load condition (684.44 ± 7.22 ms). No other main or interaction effects reached statistical significance (*ps* > 0.05).

For accuracy in the n-back task, results indicated a significant main effect of load: F_(1, 37)_ = 25.45, *p* < 0.001; η_*p*_^2^ = 0.39. Accuracy under low load (97.75 ± 0.26%) was significantly higher than that under high load (94.91 ± 0.26%). No other main or interaction effects reached statistical significance (*ps* > 0.05). These results indicate that the working memory load task was well-operationalized. Compared with the high-load condition, both athletes and non-athletes exhibited faster RTs and higher accuracy rates under low memory load, with no significant between-group differences.

#### 3.1.2 Spatial stroop task

**Figure 2:**
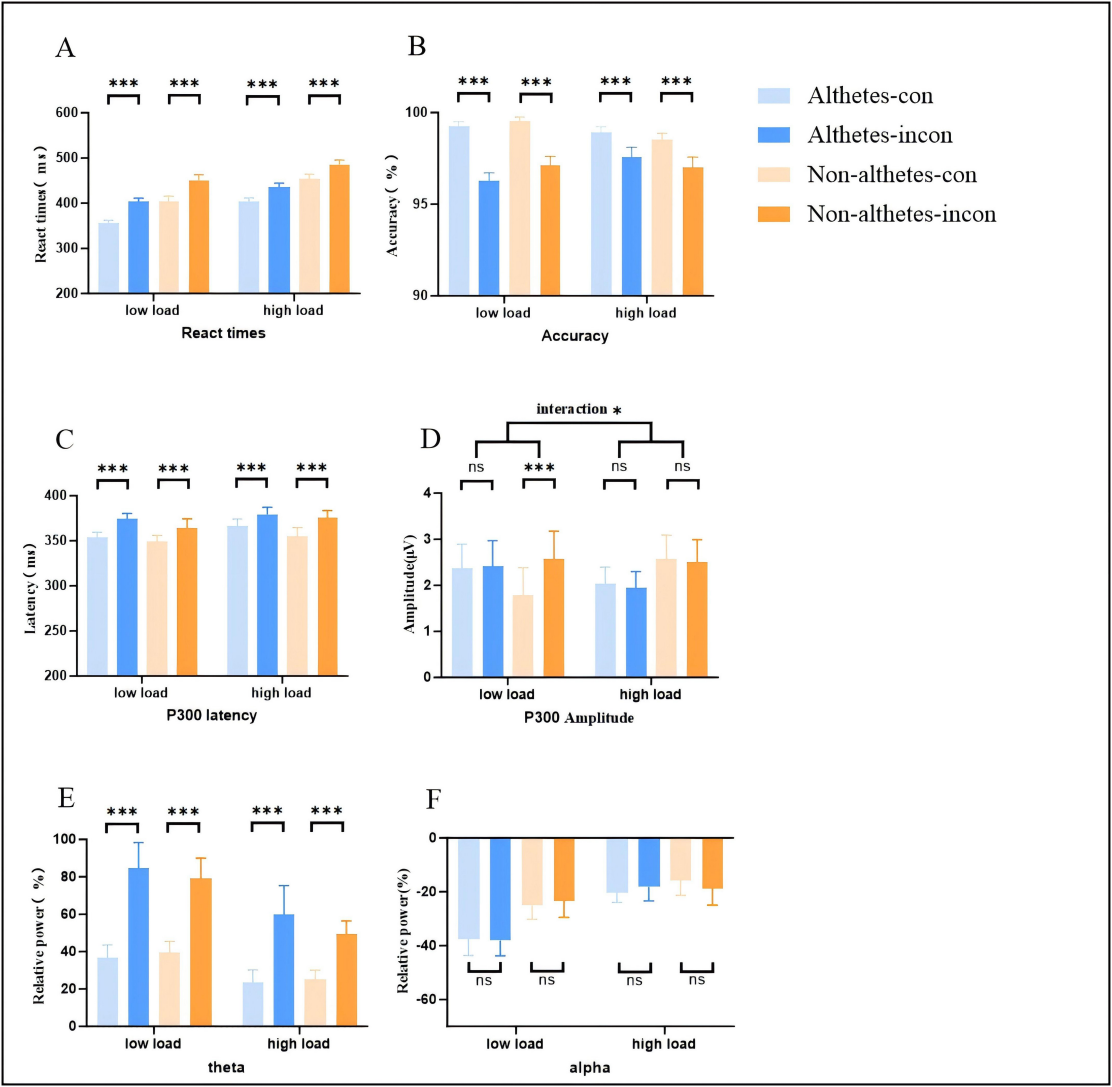
Bar plots illustrating behavioral and electrophysiological results. **(A)** Reaction times in the Spatial Stroop task. **(B)** Accuracy in the Spatial Stroop task. **(C)** P300 latency. **(D)** P300 amplitude. **(E)** Theta band power. **(F)** Alpha band power. Error bars represent the standard error of the mean (SEM). *ns.*, no significant. *p < 0.05, ***p < 0.001.

A 2 (group) × 2 (working memory load) × 2 (congruency) repeated-measures ANOVA was conducted on RTs in the Spatial Stroop task. The results revealed a significant main effect of working memory load, F_(1, 37)_ = 36.01, *p* < 0.001, η_*p*_^2^ = 0.49, with faster RTs under the low-load condition (403.68 ± 6.75 ms) than under the high-load condition (444.32 ± 6.70 ms). A significant main effect of congruency was also observed, F_(1, 37)_ = 412.44, *p* < 0.001, η_*p*_^2^ = 0.91, indicating that RTs were significantly faster in congruent trials (404.41 ± 5.53 ms) than in incongruent trials (443.59 ± 6.23 ms). In addition, a significant main effect of group was found, F_(1, 37)_ = 11.63, *p* < 0.001, η_*p*_^2^ = 0.32, with table tennis athletes responding faster (399.74 ± 7.11 ms) than non-athletes (448.26 ± 8.33 ms). Notably, the interaction between working memory load and congruency was significant, F (1, 37) = 11.53, *p* = 0.002, η_*p*_^2^ = 0.24. follow-up analyses revealed that the Stroop interference effect was significantly larger under the low-load condition (46.82 ± 6.73 ms; incongruent: 427.10 ± 7.21 ms; congruent: 380.26 ± 6.56 ms, *p* < 0.001) than under the high-load condition (30.51 ± 3.00 ms; incongruent: 460.08 ± 6.95 ms; congruent: 428.56 ± 6.82 ms, *p* < 0.001). No main or interaction effects reached statistical significance (*ps* > 0.05; see Figure 2A for detailed results).

A 2 (group) × 2 (working memory load) × 2 (congruency) repeated-measures ANOVA was conducted on accuracy in the Spatial Stroop task. The results revealed a significant main effect of working memory load, F_(1, 37)_ = 4.46, *p* = 0.041, η_*p*_^2^ = 0.10, indicating that accuracy was significantly higher under the low-load condition (98.24 ± 0.19%) than under the high-load condition (97.63 ± 0.23%). A significant main effect of congruency was also observed, F_(1, 37)_ = 39.92, *p* < 0.001, η_*p*_^2^ = 0.51, with higher accuracy in congruent trials (98.82 ± 0.12%) than in incongruent trials (97.06 ± 0.27%). No other main or interaction effects reached statistical significance (*ps* > 0.05; see Figure 2B for detailed results).

### 3.2 ERPs results

A 2 (group) × 2 (working memory load) × 2 (congruency) repeated-measures ANOVA was conducted on P300 latency. The results revealed a significant main effect of congruency, F_(1, 37)_ = 7.89, *p* < 0.001, η_*p*_^2^ = 0.37, indicating that P300 latency was significantly longer in incongruent trials (373.31 ± 5.03 ms) than in congruent trials (355.89 ± 4.37 ms). No other main or interaction effects reached statistical significance (*ps* > 0.05; see Figure 2C for detailed results).

**Figure 3:**
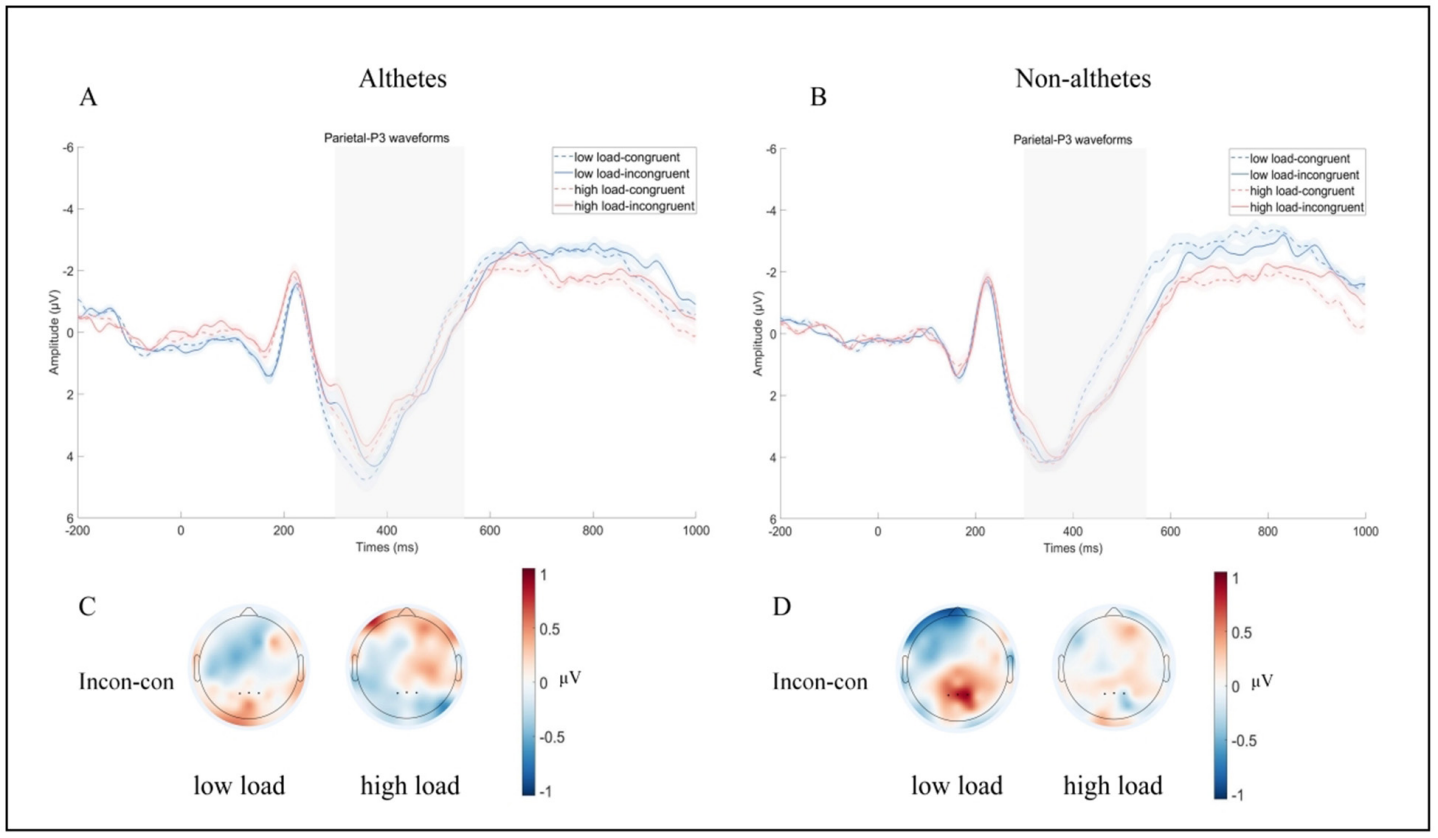
The ERP waveforms and scalp topographies for the P300 component. **(A) P300** waveforms **for the** athletes group. **(B) P300** waveforms **for the** non-athletes. **(C)** Scalp topography of P300 amplitude differences in the athletes. **(D)** Scalp topography of P300 amplitude differences in the non-athletes. The gray-shaded area indicates the time window used to calculate P300 latency and amplitude, and the shading above and below the P300 waveforms represents the 95% confidence intervals. The black dots denote the electrodes selected for P300 analysis.

A 2 (group) × 2 (working memory load) × 2 (congruency) repeated-measures ANOVA was conducted on P300 amplitude. The results revealed a significant interaction between working memory load and congruency, F _(1, 37)_ = 8.40, *p* = 0.006, η_*p*_^2^ = 0.18. follow-up analyses showed that the Stroop interference effect under the low-load condition (0.41 ± 0.14 μV; incongruent: 2.49 ± 0.40 μV; congruent: 2.08 ± 0.39 μV, *p* = 0.007) was significantly larger than that under the high-load condition (−0.08 ± 0.14 μV; incongruent: 2.22 ± 0.30 μV; congruent: 2.30 ± 0.31 μV, *p* = 0.56). Notably, a significant three-way interaction among working memory load, congruency, and group was observed for P300 amplitude, F(1, 37) = 4.37, *p* = 0.043, η_*p*_^2^ = 0.10, follow-up analyses indicated that in the non-athletes, the Stroop interference effect was significantly larger under the low-load condition (0.78 ± 0.20 μV; incongruent: 2.58 ± 0.58 μV; congruent: 1.79 ± 0.56 μV, *p* < 0.001) than under the high-load condition (−0.07 ± 0.20 μV; incongruent: 2.49 ± 0.43 μV; congruent: 2.57 ± 0.44 μV, *p* = 0.71). In contrast, in the athletes, the Stroop interference effect did not differ significantly between the low-load condition (0.04 ± 0.20 μV; incongruent: 2.41 ± 0.56 μV; congruent: 2.36 ± 0.46 μV, *p* = 0.82) and the high-load condition (−0.09 ± 0.19 μV; incongruent: 1.94 ± 0.41 μV; congruent: 2.03 ± 0.43 μV, *p* = 0.64). No other main or interaction effects reached statistical significance (*ps* > 0.05; see Figures 2D, 3 for detailed results).

### 3.3 ERSPs results

#### 3.3.1 Theta band

**Figure 4:**
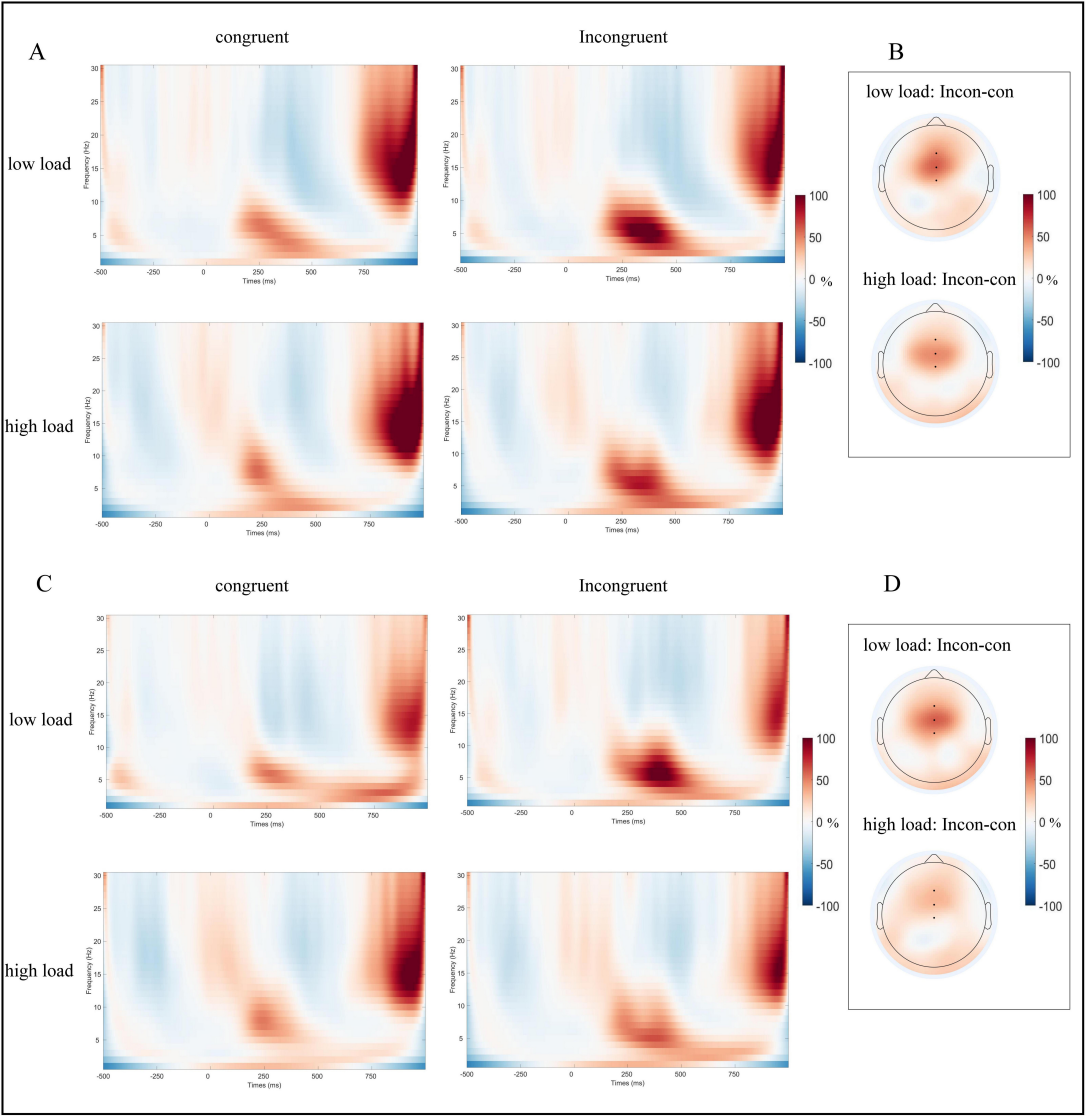
ERSPs results for the theta band. **(A)** Time–frequency representations of theta band under low- and high-load conditions for congruent and incongruent trials in athletes. **(B)** Scalp topographies of theta band power differences between low- and high-load conditions in athletes. **(C)** Time–frequency representations of theta band under low- and high-load conditions for congruent and incongruent trials in non-athletes. **(D)** Scalp topographies of theta band power differences between low- and high-load conditions in the non-athletes. Black dots indicate the electrodes selected for theta band analysis.

A 2 (group) × 2 (working memory load) × 2 (congruency) repeated-measures ANOVA was conducted on the theta band. The results revealed a significant main effect of working memory load, F_(1, 37)_ = 15.12, *p* < 0.001, η_*p*_^2^ = 0.29, indicating that theta band power was significantly greater under the low-load condition (0.58 ± 0.06) than under the high-load condition (0.39 ± 0.05). A significant main effect of congruency was also observed, *F*_(1, 37)_ = 48.43, *p* < 0.001, η_*p*_^2^ = 0.54, with higher theta band power in incongruent trials (0.68 ± 0.07) than in congruent trials (0.29 ± 0.04). Importantly, a significant interaction between working memory load and congruency was found, *F*_(1, 37)_ = 7.58, *p* = 0.009, η_*p*_^2^ = 0.17, follow-up analyses analyses revealed that the Stroop interference effect under the low-load condition (0.47 ± 0.07; incongruent: 0.81 ± 0.08; congruent: 0.34 ± 0.04, *p* < 0.001) was significantly larger than that under the high-load condition (0.30 ± 0.05; incongruent: 0.54 ± 0.08; congruent: 0.24 ± 0.04, *p* < 0.001). No other main or interaction effects reached statistical significance (*ps* > 0.05; see Figures 2E, 4 for detailed results).

#### 3.3.2 Alpha band

**Figure 5:**
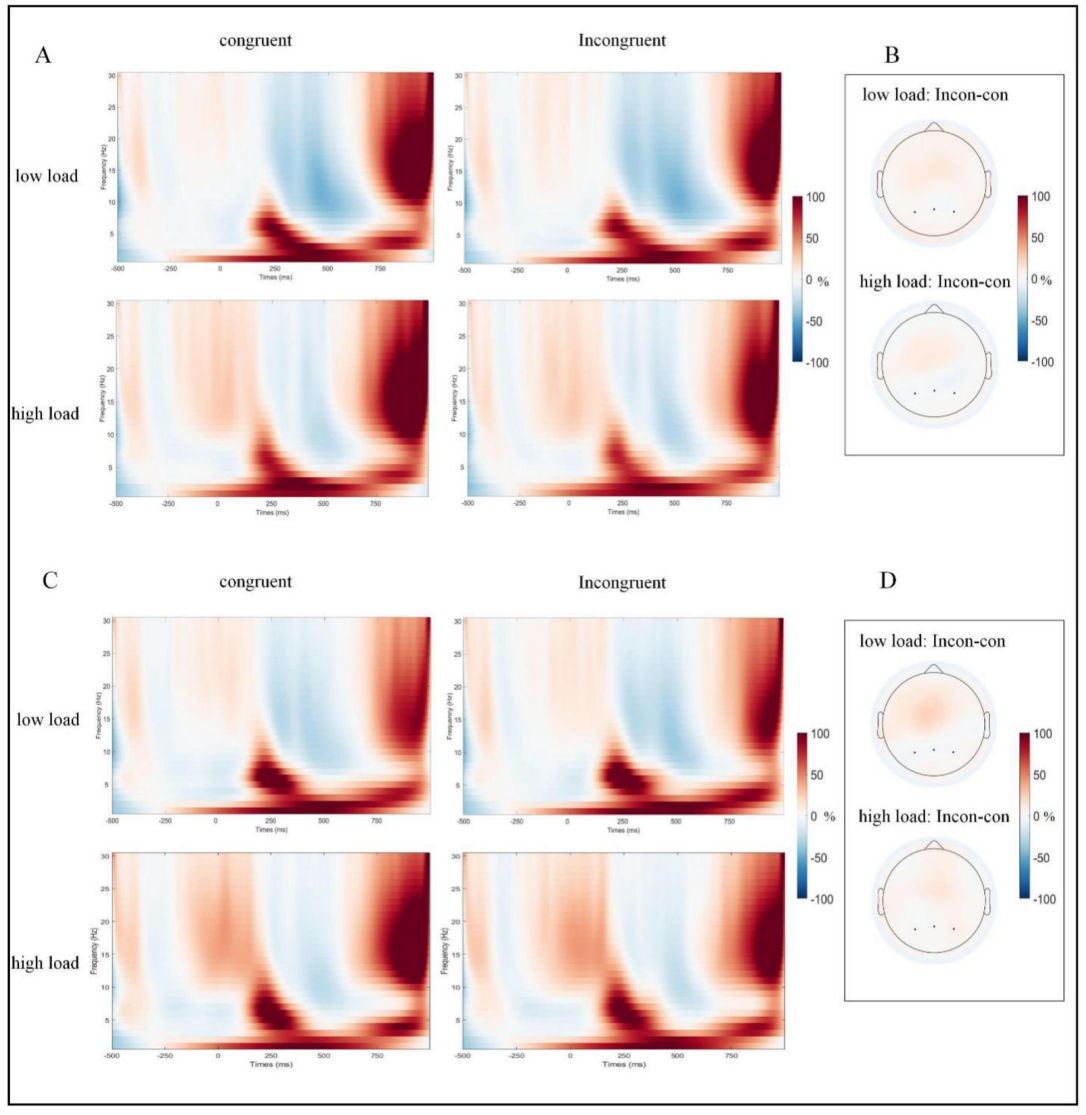
ERSPs results for the alpha band. **(A)** Time–frequency representations of the alpha band under low- and high-load conditions for congruent and incongruent trials in athletes. **(B)** Scalp topographies of alpha band power differences between low- and high-load conditions in athletes. **(C)** Time–frequency representations of the alpha band under low- and high-load conditions for congruent and incongruent trials in non-athletes. **(D)** Scalp topographies of alpha band power differences between low- and high-load conditions in the non-athletes. Black dots indicate the electrodes selected for alpha band analysis.

A 2 (group) × 2 (working memory load) × 2 (congruency) repeated-measures ANOVA was conducted on the alpha band. The results revealed a significant main effect of working memory load, F_(1, 37)_ = 22.26, *p* < 0.001, η_*p*_^2^ = 0.37, indicating that alpha band power was significantly greater under the low-load condition (−0.31 ± 0.03) than under the high-load condition (−0.18 ± 0.03). No other main or interaction effects reached statistical significance (*ps* > 0.05; see Figures 2F, 5 for detailed results).

## 4 Discussion

The present study employed a dual-task paradigm combining an n-back task and a Spatial Stroop task, recruiting table tennis athletes and non-athletes, by integrating ERPs and ERSPs analyses, the study explored the effect of working memory load on interference inhibition and whether motor expertise moderates this relationship.

The results showed that (1) increased working memory load reduced the impact of distraction, leading to reduced Stroop interference effect; (2) a significant three-way interaction among working memory load, congruency, and group was observed for P300 amplitude, indicating that the Stroop interference effect on P300 amplitude decreased with increasing load in non-athletes, whereas this pattern was not evident in table tennis athletes. These findings are discussed in the subsequent sections.

### 4.1 The effect of working memory load on interference inhibition in table tennis athletes

The study found that, as working memory load increased, both table tennis athletes and non-athletes exhibited reduced susceptibility to Spatial location interference in the Spatial Stroop task, as reflected by a decrease in the Stroop interference effect. This finding is consistent with our hypotheses. Moreover, compared with previous studies employing single-task paradigms (Bayramova et al., 2021; Scharinger et al., 2015), the current dual-task design similarly demonstrated that increased working memory load reduces Stroop interference effect. This phenomenon is likely related to the efficient engagement of the fronto-parietal networks induced by the n-back task (Kim et al., 2017). Neuroimaging evidence has shown that the n-back task robustly activates a fronto-parietal network centered on the dorsolateral prefrontal cortex, which serves as a shared neural substrate for working memory and inhibitory control. Enhanced activation within this network is thought to reflect improved attentional control and top-down regulation (Glahn et al., 2002; Meidenbauer et al., 2021; Tomasi et al., 2007; Miyake et al., 2000). Therefore, the present findings provide further support for the task-engagement/distraction trade-off (TEDTOFF) model, thereby deepening our understanding of the interaction between working memory and interference inhibition.

Consistent with several previous studies (Bayramova et al., 2021; Güldenpenning et al., 2020; Scharinger et al., 2015), the behavioral results indicated that increased working memory load reduced the Stroop interference effect on RTs, whereas the accuracy-based Stroop interference effect remained unaffected by load. This dissociation may be attributed to the relatively low difficulty of the Spatial Stroop task, which yielded uniformly high accuracy (>95%) across all conditions and led to a pronounced ceiling effect. Future studies could address this limitation by introducing time pressure or employing more demanding interference-inhibition tasks, thereby allowing a more sensitive assessment of load-related effects on the accuracy-based Stroop interference effect.

However, when comparing the table tennis athletes and non-athletes, a significant main effect of group was observed only for RTs, with table tennis athletes exhibiting shorter overall RTs than non-athletes. This advantage may be attributable to enhanced perceptual sensitivity to Spatial information developed through long-term sport-specific training. Nevertheless, no reduced Stroop interference effect on RTs was observed in the athletes, indicating that they did not demonstrate a superior inhibitory advantage in response speed when processing distractors. This finding is inconsistent with the results reported by (Huang et al. 2024), which may be explained by differences in task-stimulus properties across studies. Specifically, (Huang et al. 2024) employed word-based stimuli in their Spatial Stroop task, whereas the present study used arrow stimuli. According to the stimulus–response compatibility classification model (Kornblum and Lee, 1995; Kornblum et al., 1999), arrow stimuli involve a high degree of overlap among stimulus direction, Spatial location, and response dimensions. Compared with word stimuli that require semantic encoding, arrow stimuli elicit stronger automatic response tendencies and greater distraction, leading to similar levels of interference across both groups. This suggests that when using cognitive tasks to assess athletes' expert advantage in future studies, careful consideration must be given to the intrinsic properties of task stimuli and the potential intensity of interference they may induce.

The electrophysiological findings reveal the neural activity characteristics of the fronto-parietal network underlying the effect of working memory load on interference inhibition. The theta band is widely regarded as a key neural marker of prefrontal cognitive control processes (Cavanagh and Frank, 2014; Haciahmet et al., 2023). In the study, midfrontal theta band power was examined within the 250–500 ms time window, and a significant increase in theta band power was observed under the incongruent relative to the congruent condition in the Spatial Stroop task. This finding suggests that participants were required to engage enhanced cognitive control in the prefrontal region at an early stage of conflict monitoring to resolve distractors. (Zhao et al. 2014) reported that the theta band power interference effect in the Stroop task is significantly modulated by working memory load, with source localization analyses indicating that theta activities originate from the left middle frontal gyrus, a region known to play a critical role in the neural mechanisms of inhibitory control. Accordingly, the reduction in theta band power interference effect observed in the present study likely reflects a diminished impact of distractors under higher working memory load, resulting in reduced demands on midfrontal cognitive control mechanisms. Notably, no significant differences in theta-band power were observed between table tennis athletes and non-athletes, suggesting that long-term sport-specific training may have limited effects on enhancing prefrontal cognitive control mechanisms at the level indexed by theta activity (Wang et al., 2017).

This study found a significant reduction in alpha band power in the parietal region under high working memory load, reflecting enhanced functional inhibition of task-irrelevant brain regions (Klimesch et al., 2007; Klimesch, 2012). However, no significant interference effect was observed, which is inconsistent with the findings of (Scharinger et al. 2015). Differences may stem from the experimental design: compared to Scharinger's single-task design, this study employed a dual-task design, imposing greater cognitive demands on participants. Additionally, research indicates that sustained updating of working memory load may impact the alpha band's specific response to distractions (Lenartowicz et al., 2021), potentially explaining the lack of a significant alpha power interference effect in this study. Furthermore, no significant intergroup differences were observed in the alpha band, suggesting that table tennis athletes and non-athletes share similar attentional control mechanisms in posterior brain regions.

### 4.2 The moderating role of motor expertise in P300 amplitude

The P300 component is widely regarded as a neural marker of endogenous attention, with its latency and amplitude reflecting task-related processing speed and attentional resource allocation, respectively (Magliero et al., 1984; Polich and Kok, 1995; Polich, 2007). In the present study, the interaction between working memory load and congruency emerged only in P300 amplitude, but not in latency, suggesting that the effect of working memory load on interference inhibition is primarily reflected in attentional resource allocation rather than stimulus processing speed. More importantly, the present study identified a moderating role of motor expertise in this attentional resource allocation process. The significant three-way interaction among load, congruency, and group indicated that a load-by-congruency interaction was evident in non-athletes, such that the P300 amplitude Stroop interference effect decreased significantly with increasing load (low-load interference effect: 0.78 ± 0.20 μV; high-load interference effect: −0.07 ± 0.20 μV), reflecting that non-athletes required greater attentional resource investment to resolve distractor interference and that this investment was modulated by working memory load. In contrast, the P300 amplitude the Stroop interference effect in table tennis athletes was insensitive primarily to load manipulation (low load: 0.04 ± 0.20 μV; high load: −0.09 ± 0.19 μV), indicating a more efficient and stable pattern of attentional resource allocation under working memory load, consistent with characteristics of neural efficiency (Huang et al., 2024; Li and Smith, 2021; Neubauer and Fink, 2009).

From a neural mechanism perspective, the moderating role of motor expertise may originate from adaptive structural and functional brain changes induced by long-term specialized training (Vints et al., 2022). Previous studies have shown that athletes are able to develop more focused and efficient task-related neural networks, and that the percentage amplitude of low-frequency fluctuations in the postcentral gyrus is negatively correlated with years of sports training, reflecting training-induced functional reorganization and enhanced processing efficiency (Peng et al., 2025; Wu et al., 2022). In addition, resting-state studies have found that table tennis athletes exhibit lower local spontaneous activity and static functional connectivity in brain regions associated with visuomotor transformation, suggesting that key processing stages may be more automated and require fewer baseline neural resources (Li et al., 2023).

Notably, the present study did not observe similar moderating patterns in theta or alpha band power, suggesting that the cognitive benefits conferred by long-term specialized training may be relatively limited for cognitive control mechanisms indexed by midfrontal theta and for inhibitory states and attentional gating processes indexed by posterior alpha. Instead, prior research has suggested that sports training may act as a stressor that activates the cerebellar–locus coeruleus–norepinephrine (LC–NE) system, thereby facilitating neural inhibition and resource allocation required for task execution; such physiological effects are more likely to be reflected in modulations of P300 amplitude (Kao et al., 2025). Therefore, the present findings indicate that the moderating role of motor expertise is component-specific, with primary effects observed at the P300 amplitude level. On the one hand, the present findings clarify the specific role of motor expertise as a domain-specific cognitive difference within the TEDTOFF model, thereby extending its applicability to expert athletes. On the other hand, these results provide novel electrophysiological evidence supporting the view that long-term specialized training promotes brain development. Future research may consider table tennis training as a targeted non-pharmacological intervention to improve cognitive performance in individuals with impaired inhibitory control or specific neurological dysfunctions.

Although the present study provides several valuable findings, some limitations should be acknowledged. First, the sample size of the present study was relatively small, which may have introduced selection bias and increased the influence of individual differences. Therefore, future studies should recruit larger samples and may include athletes from closed-skill sports to examine further the moderating effects of different types of motor expertise on the relationship between working memory load and interference inhibition. In addition, the present study primarily relied on electroencephalography to investigate neural activities in frontal and parietal regions. Although EEG provides high temporal resolution, its limited spatial resolution constrains our ability to capture interactions among broader brain networks. Future studies may adopt multimodal neuroimaging techniques, such as functional magnetic resonance imaging (fMRI) and magnetoencephalography (MEG), to more precisely elucidate the spatiotemporal neural mechanisms underlying the interaction between working memory and interference inhibition.

## 5 Conclusion

The present study demonstrated that increased working memory load reduces the impact of distractors, as reflected by reduced Stroop interference effect on RTs and midfrontal theta band power, as well as decreased parietal alpha band power. These effects may be attributed to the efficient engagement of the fronto-parietal network induced by the n-back task, giving rise to an attention-enhancement cognitive mode. More importantly, the present study revealed that motor expertise moderated the effect of working memory load on interference inhibition, exclusively at the P300 amplitude level. Compared with non-athletes, table tennis athletes exhibited a more efficient and stable pattern of attentional resource allocation under working memory load, reflecting the component-specific characteristic of the moderating role of motor expertise. Overall, the present findings extends current understanding of the interaction between working memory load and interference inhibition in expert athletes and provide electrophysiological evidence supporting the role of long-term specialized training in promoting brain function.

## Data Availability

The original contributions presented in the study are included in the article/supplementary material, further inquiries can be directed to the corresponding author.
