# Correction: The effect of working memory load on interference inhibition in table tennis athletes: the moderating role of motor expertise

**DOI:** 10.3389/fpsyg.2026.1872727

**Published:** 2026-06-10

**Authors:** Hongyu Chen

**Affiliations:** College of Physical Education and Health, East China Normal University, Shanghai, China

**Keywords:** interference effect, interference inhibition, motor expertise, table tennis athletes, working memory load

There was a mistake in Figure 1 as published. [Technical error in the creation of Figure 1B]. The corrected Figure 1 and Figure 1B appears below.

**Figure 1:**
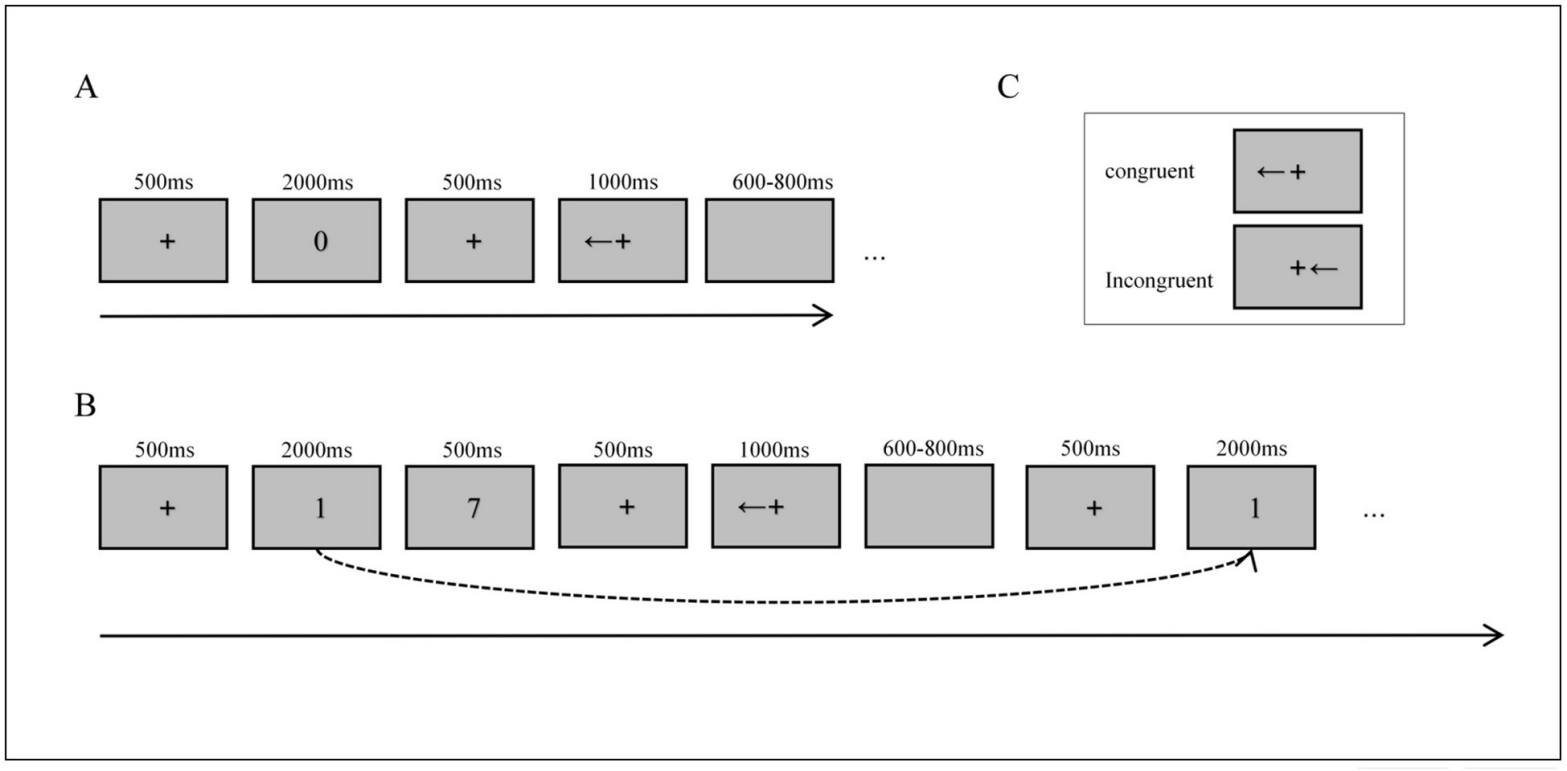
Schematic illustration of experimental trial structures and stimulus types. **(A)** Structure of the dual-task under low-load (0-back) condition. **(B)** Structure of the dual-task under high-load (2-back) condition. **(C)** Schematic of the Spatial Stroop task under congruent and incongruent conditions.

There was a mistake in Figure 5C as published. [Technical error in the creation of Figure 5C]. The corrected Figure 5 and Figure 5C appears below.

**Figure 5:**
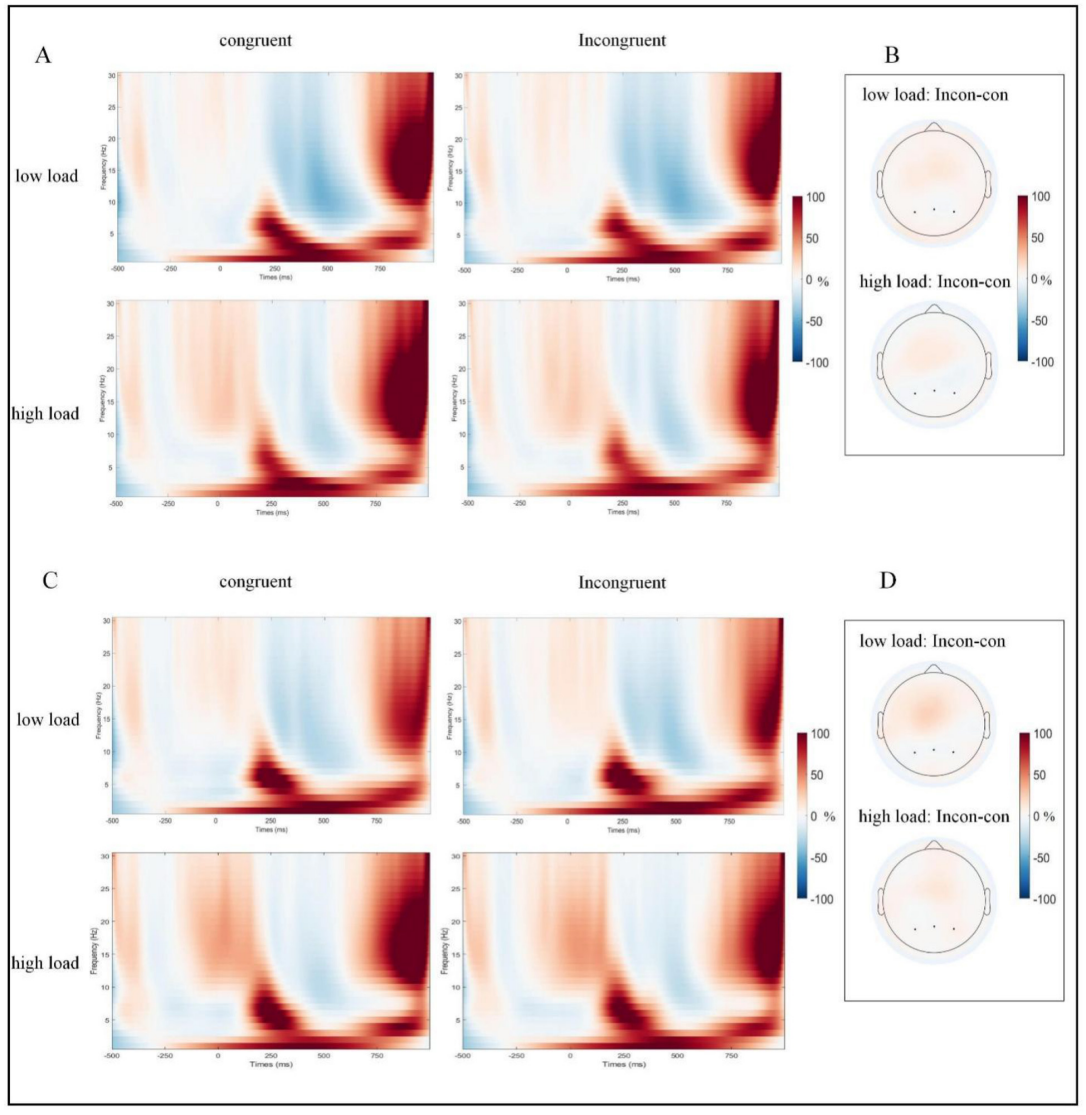
ERSPs results for the alpha band. **(A)** Time–frequency representations of the alpha band under low- and high-load conditions for congruent and incongruent trials in athletes. **(B)** Scalp topographies of alpha band power differences between low- and high-load conditions in athletes. **(C)** Time–frequency representations of the alpha band under low- and high-load conditions for congruent and incongruent trials in non-athletes. **(D)** Scalp topographies of alpha band power differences between low- and high-load conditions in the non-athletes. Black dots indicate the electrodes selected for alpha band analysis.

The original version of this article has been updated.

